# Development and validation of a deep learning model to predict heparin response in critically ill patients with deep vein thrombosis receiving continuous intravenous heparin infusion

**DOI:** 10.1097/BS9.0000000000000300

**Published:** 2026-08-17

**Authors:** Shengjun Liu, Longxiang Su, Sihang Zhang, Huacong Cai, Weiling Shou, Bo Tang, Xinchen Wang, Anhui Guo, Weiguo Zhu, Yun Long

**Affiliations:** a Department of Critical Care Medicine, State Key Laboratory of Complex Severe and Rare Diseases, Peking Union Medical College Hospital, Chinese Academy of Medical Sciences & Peking Union Medical College, Beijing, China; b Goodwill Information Technology Co., Ltd., Beijing, China; c Department of Hematology, Peking Union Medical College Hospital, Chinese Academy of Medical Sciences & Peking Union Medical College, Beijing, China; d Department of Clinical Laboratory, Peking Union Medical College Hospital, Chinese Academy of Medical Sciences & Peking Union Medical College, Beijing, China; e Department of General Medicine, Peking Union Medical College Hospital, Peking Union Medical College, Chinese Academy of Medical Sciences

**Keywords:** Activated partial thromboplastin time, Deep learning, Deep vein thrombosis, Heparin

## Abstract

Continuous intravenous heparin infusion is widely used for deep vein thrombosis (DVT) in the intensive care unit (ICU), but accurate prediction of activated partial thromboplastin time (APTT) remains challenging due to patient heterogeneity and complex drug responses. A deep learning model was developed using heparin administration data, laboratory tests, and patient history to predict future APTT values in ICU patients with DVT. Data from 796 patients receiving continuous heparin infusion were collected, including demographics, comorbidities, treatment, and laboratory information, with external validation performed on 514 patients from the Medical Information Mart for Intensive Care (MIMIC) database. A 2-layer Long Short-Term Memory model with dropout was trained and evaluated. Optimal performance was achieved with the [*t* − 12, *t* − 2] window, yielding a mean absolute error of 1.885, root mean square error of 4.732, *R*^2^ of 0.945, and mean absolute percentage error of 4.433. Subgroup analyses demonstrated robust accuracy across critical APTT zones and therapeutic categories (subtherapeutic, therapeutic, supratherapeutic), with area under the curve values of 0.92, 0.81, and 0.92. The model predicted abnormal APTT values a mean of 6 hours in advance and could improve guidance over physician-led management in approximately 70% of cases. External validation confirmed good generalizability. Feature importance identified the difference between 45 s and APTT, cumulative heparin dose, and infusion rate as the leading predictors. Collectively, this deep learning model accurately forecasts future APTT values in critically ill patients with DVT receiving intravenous heparin, supporting a shift from reactive to proactive, data-driven heparin management in the ICU with potential benefits for patient safety and treatment efficacy.

## 1. INTRODUCTION

Deep vein thrombosis (DVT) is a prevalent and serious vascular disorder that affects millions worldwide and represents a substantial public health burden. According to the U.S. Centers for Disease Control and Prevention, approximately 900,000 individuals in the United States are affected by venous thromboembolism (VTE), which includes both DVT and pulmonary embolism, each year. VTE is associated with an estimated 60,000 to 100,000 deaths annually in the United States, and many survivors experience long-term complications.^1^ In the intensive care unit (ICU), DVT is particularly common and carries a high risk of progression to pulmonary embolism if timely and effective anticoagulation therapy is not administered. Unfractionated heparin (UFH) is a standard treatment for DVT, and activated partial thromboplastin time (APTT) is a critical laboratory measure for monitoring the efficacy and safety of UFH therapy.^2,3^ Although APTT can be readily measured in hospital settings, predicting the APTT response during UFH treatment remains clinically important because of UFH’s variable pharmacokinetics and the time-sensitive need to achieve therapeutic anticoagulation. The anticoagulant effect of UFH varies widely among patients because of factors such as body weight, liver function, and plasma protein binding, leading to heterogeneous APTT responses.^4–6^ Frequent APTT testing, typically every 6 hours initially, provides snapshots but does not anticipate how levels may change between tests or after dose adjustments. Delayed achievement of a therapeutic APTT increases the risk of recurrent DVT during the first few days of treatment, whereas excessive dosing increases bleeding risk. By forecasting APTT, clinicians may tailor initial and subsequent doses more precisely, minimize trial-and-error adjustments, and improve outcomes in time-critical DVT management.

Existing tools, such as clinical risk scores or pharmacokinetic models, often lack the granularity and adaptability needed to predict APTT with high precision in dynamic ICU settings.^7^ These limitations highlight the need for more sophisticated approaches that can integrate real-time patient data and treatment responses. Recent advancements in artificial intelligence (AI), particularly deep learning, have shown promise for processing complex clinical data and generating accurate predictions.^8–10^ However, limited studies have explored time-dependent machine learning approaches to predict APTT based on heparin dosing regimens and laboratory parameters. Two prior studies have attempted to predict APTT in clinical settings. In both studies, the models were not specific to patients with DVT and did not incorporate heparin administration data. Both studies also had limitations, including high root mean square error (RMSE) or a lack of external validation. Given these gaps, there remains an urgent need for additional research focused specifically on patients with DVT, supported by robust external validation.^11,12^

In this study, we propose a novel deep learning model trained on real-world data from ICU patients receiving heparin therapy for DVT. Our approach leverages detailed heparin administration information, including infusion rates and cumulative doses over the preceding several hours, as well as laboratory test results, to predict APTT over the subsequent few hours. By incorporating temporal dependencies in heparin dosing patterns and physiological changes, our model aims to provide accurate and personalized APTT predictions. This advancement could enable more precise titration of anticoagulant therapy, thereby reducing the risk of both bleeding and thrombotic complications. Furthermore, external validation using an independent dataset from the Medical Information Mart for Intensive Care (MIMIC) database supports the generalizability and robustness of the model.

## 2. METHODS

### 2.1. Study design

**Figure 1.**
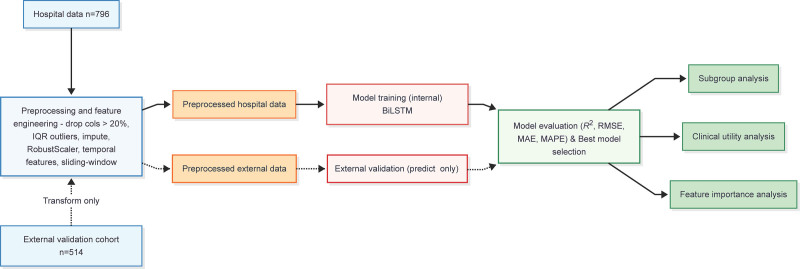
The overall study flow. IQR = interquartile range, LSTM = long short-term memory, MAE = mean absolute error, MAPE = mean absolute percentage error, *R*^2^ = coefficient of determination, RMSE = root mean square error.

This retrospective observational study aimed to develop a deep learning model using heparin administration data, laboratory test results, and medical history to predict APTT values in patients with DVT admitted to the ICU, thereby improving the optimization of DVT treatment. First, deep learning regression models with different time windows ([*t* − 12, *t* − 10], [*t* − 12, *t* − 9], …, [*t* − 12, *t* − 2], where *t* denotes the APTT measurement time) were developed and evaluated to predict APTT, and the best-performing model was selected for subsequent analyses. Subgroup analyses were conducted to assess model performance across categories, including critical vs noncritical ranges, subtherapeutic vs therapeutic vs supratherapeutic ranges, and high-risk vs medium-risk vs low-risk error categories. In addition, the clinical utility of the model was evaluated by calculating the average lead time of alerts for abnormal APTT values, particularly for subtherapeutic and supratherapeutic values. Feature importance analysis was performed to identify key variables contributing to the model’s predictions. To evaluate generalizability, external validation was conducted using a dataset from the MIMIC-IV database.^13,14^ The overall workflow is illustrated in Figure 1. The study protocol was approved by the Ethics Review Board of Peking Union Medical College Hospital (I-25PJ3176). All patient data were anonymized and deidentified before analysis. The requirement for informed consent was waived because of the retrospective nature of the study. All procedures adhered to the Declaration of Helsinki and applicable ethical guidelines for medical research.

### 2.2. Data sources and patient inclusion

Clinical records of 845 patients with DVT receiving continuous intravenous heparin infusion in the ICU from January 2013 to December 2022 were initially included. The inclusion criteria were as follows: diagnosis of DVT by ultrasonography, age >18 years, receipt of heparin therapy for at least 24 hours, and at least 3 APTT measurements from the start of heparin therapy to 12 hours after treatment. Patients were excluded if the heparin infusion rate or body weight was not recorded. For external validation, matched records were extracted from the MIMIC-IV database, a freely accessible relational database containing deidentified patient data from Beth Israel Deaconess Medical Center.

### 2.3. Data collection

Data were collected from 2 sources: the ICU database at Peking Union Medical College Hospital and the MIMIC-IV database. Collected variables included demographic characteristics, comorbidities, laboratory values, heparin infusion rate, intravenous heparin bolus dose, cumulative heparin dose, timestamps for laboratory measurements, and timestamps for heparin administration. All personal identifiers were removed to ensure patient confidentiality. Shengjun Liu (Record ID: 76247108) was authorized to access the MIMIC database and oversaw data extraction. Detailed data collection procedures for MIMIC-IV are provided in the Supplementary Material, https://links.lww.com/BS/A172, https://links.lww.com/BS/A173, and units of measurement were harmonized between the hospital and MIMIC-IV datasets as required.

### 2.4. Data preprocessing

Laboratory parameters, heparin infusion rate, intravenous heparin bolus dose, and cumulative heparin dose within each time window ([*t* − 12, *t* − 10], [*t* − 12, *t* − 9], …, [*t* − 12, *t* − 2], where *t* denotes the APTT measurement time) were extracted and used for subsequent preprocessing. To improve model performance, engineered features were created, including cyclical temporal features (Hour_sin and Hour_cos), a time-delta feature (Time_since_first), and a statistical aggregation feature (Gap_between_45_APTT). Hour_sin and Hour_cos encode the hour of day as continuous sinusoidal values, thereby preserving temporal proximity between 23:59 and 00:01. Time_since_first captures the elapsed time in seconds since the first APTT measurement in each ICU stay. Gap_between_45_APTT quantifies the deviation of the most recent APTT value from the therapeutic upper limit of 45 seconds. Outliers were identified using a Tukey-style interquartile range (IQR) rule (values < *Q*1 − 5 × IQR or > *Q*3 + 5 × IQR) and removed. Variables with > 20% missingness were excluded, and remaining missing values were imputed using iterative imputation (IterativeImputer). All features were standardized using RobustScaler. Finally, APTT values >100 seconds were capped at 100 seconds, and a log transformation was applied during training to mitigate right skewness; the transformation was subsequently inverted to return predictions to the original scale.

### 2.5. Model development and training

Deep learning models based on long short-term memory (LSTM) networks were developed for time-series prediction. Before training, the data were formatted as sequences suitable for LSTM input, which involved capturing serial measurements of laboratory values (eg, international normalized ratio [INR] and fibrinogen) and heparin infusion rates at regular intervals. Missing data points were addressed using appropriate imputation methods to maintain data completeness. Variables were then normalized to a consistent scale, and relevant features capturing temporal patterns were extracted for LSTM modeling. The model architecture consisted of an LSTM layer with a hidden state size of 128 units to capture temporal dependencies, a dropout layer with a rate of 0.5 to mitigate overfitting, a second LSTM layer with 128 units to process the output of the previous layer, a second dropout layer with a rate of 0.5, a dense hidden layer with 4 units and rectified linear unit activation, and a final dense layer with 1 unit and linear activation for scalar output. The model was trained using the Adam optimizer for 1000 epochs with a learning rate of 0.001 and a batch size of 32. Early stopping was implemented to reduce overfitting by terminating training if no improvement in validation loss occurred for 20 consecutive epochs. Linear regression and XGBoost models were trained as baseline comparators.

### 2.6. Model evaluation and subgroup analysis

Model performance was evaluated using mean absolute error (MAE), RMSE, coefficient of determination (*R*^2^), and mean absolute percentage error (MAPE). The formulas for these evaluation metrics are presented below. In these expressions, *y_i_* denotes the *i*-th true APTT, ${\hat{y}}_{i}$ is the *i*-th predicted APTT value, *ȳ* denotes the mean of the observed APTT values, and n denotes the total number of samples. Models using different time windows yielded different MAE, RMSE, *R*^2^, and MAPE values, and the best-performing model was selected as the final model for subsequent analyses. APTT measurements were categorized into a critical range (APTT <35 seconds or APTT >45 seconds) and a noncritical range (APTT ∈ [35, 45] seconds). The distribution of absolute prediction errors in each range was analyzed using density curves. Predictions were classified as high-risk errors if the observed APTT was outside the therapeutic range (APTT ∈ [35, 45] seconds) but the predicted value was within it; medium-risk errors if the observed APTT was within the therapeutic range but the predicted value was outside it; and low-risk errors otherwise. The proportions of these error types were calculated. APTT values were also grouped as subtherapeutic (APTT <35 seconds), therapeutic (APTT ∈ [35, 45] seconds), or supratherapeutic (APTT >45 seconds) based on the thresholds of 35 and 45 seconds. Model performance in distinguishing these groups was evaluated using confusion matrices and receiver operating characteristic (ROC) curves, with the area under the ROC curve (AUC) reported.


$$\begin{array}{c} MAE=\frac{1}{n}\underset{i=1}{\overset{n}{\sum }}\;|y_{i}-{\hat{y}}_{i}|RMAE=\sqrt{\frac{1}{n}\underset{i=1}{\overset{n}{\sum }}\;{\left(y_{i}-{\hat{y}}_{i}\right)}^{2}} \end{array}$$

$$\begin{array}{c} R^{2}=1\text{-}\frac{\sum_{i=1}^{n}{\left(y_{i}-{\hat{y}}_{i}\right)}^{2}}{\sum_{i=1}^{n}{\left(y_{i}-\bar{y}\right)}^{2}}MAPE=\frac{100\%}{n}\underset{i=1}{\overset{n}{\sum }}\;|\frac{y_{i}-{\hat{y}}_{i}}{y_{i}}| \end{array}$$

### 2.7. Clinical value assessment

To evaluate the model’s ability to provide early warnings for abnormal APTT values, we analyzed the lead time, defined as the number of hours in advance that the model could predict deviations from the therapeutic range. For APTT records in which the model predicted impending subtherapeutic or supratherapeutic values, we assessed whether heparin dose adjustments were appropriate by calculating the proportion of correct adjustments (eg, the model predicted subtherapeutic APTT and clinicians increased the heparin infusion rate), the proportion of no adjustment (eg, the model predicted subtherapeutic APTT but clinicians did not change the infusion rate), and the proportion of incorrect adjustments (eg, the model predicted subtherapeutic APTT but clinicians decreased the infusion rate). In addition, the absolute change in APTT from the alarm time to the event time was calculated and compared across these 3 groups. These analyses provided insights into the model’s utility for guiding clinical decision-making regarding heparin dose titration.

### 2.8. Feature importance analysis and generalizability assessment

SHapley Additive exPlanations were used to identify the most important features and quantify their relative contributions. To assess model generalizability, external validation was performed using an independent MIMIC-IV dataset, and performance was evaluated using MAE, RMSE, *R*^2^, and MAPE.

### 2.9. Statistics

Continuous variables are presented as the mean ± standard deviation (SD) for normally distributed data or as the median (IQR) for non-normally distributed data. Categorical variables are presented as counts and percentages. Comparisons between groups were performed using the independent-samples *t* test for normally distributed variables and the Mann–Whitney *U* test for non-normally distributed variables. A 2-sided *p* value <0.050 was considered statistically significant. The study flowchart was created using Mermaid, and all analyses and visualizations were conducted using Python 3.12.

## 3. RESULTS

### 3.1. Patient characteristics

A total of 845 patients were initially included; 49 were excluded because heparin infusion data or body weight were not recorded. Consequently, 796 patients were included in the final analysis and were randomly split into training and test sets. For external validation, 514 matched patients were extracted from the MIMIC-IV database. Table 1 presents demographic and clinical characteristics across 3 datasets (training, test, and external validation), with sample sizes of 599, 197, and 514, respectively. The mean age ranged from 57.6 to 63.9 years (SD, 13.8–16.2), mean height ranged from 166.8 to 170.2 cm (SD, 7.8–10.5), and mean weight ranged from 68.8 to 89.2 kg (SD, 13.7–26.3). The proportion of male patients ranged from 57.8% to 59.6%, whereas the proportion of female patients ranged from 40.4% to 42.2%. Comorbidities varied across datasets and included cardiovascular disease (79.4%–86.3%), metabolic disease (37.1%–57.8%), cancer (32.7%–43.1%), kidney disease (50.8%–58.6%), respiratory disease (72.5%–87.5%), hematologic and immune disease (7.6%–35.8%), infection (57.2%–70.6%), surgery (8.9%–50.4%), and liver disease (46.1%–50.8%).

**Table 1:** Patient characteristics.

	Overall	Training set	Test set	External validation set
Patient number, n	1310	599	197	514
Age, years, mean (SD)	61.2 (15.1)	63.9 (13.8)	61.9 (14.0)	57.6 (16.2)
Height, cm, mean (SD)	168.1 (9.2)	167.2 (8.5)	166.8 (7.8)	170.2 (10.5)
Weight, kg, mean (SD)	76.9 (21.8)	68.8 (13.7)	69.6 (11.7)	89.2 (26.3)
Gender, n (%)				
Female	539 (41.1)	242 (40.4)	80 (40.6)	217 (42.2)
Male	771 (58.9)	357 (59.6)	117 (59.4)	297 (57.8)
Cardiovascular disease, n (%)				
No	244 (18.6)	111 (18.5)	27 (13.7)	106 (20.6)
Yes	1066 (81.4)	488 (81.5)	170 (86.3)	408 (79.4)
Metabolic disease, n (%)				
No	698 (53.3)	357 (59.6)	124 (62.9)	217 (42.2)
Yes	612 (46.7)	242 (40.4)	73 (37.1)	297 (57.8)
Cancer, n (%)				
No	804 (61.4)	341 (56.9)	117 (59.4)	346 (67.3)
Yes	506 (38.6)	258 (43.1)	80 (40.6)	168 (32.7)
Kidney disease, n (%)				
No	591 (45.1)	281 (46.9)	97 (49.2)	213 (41.4)
Yes	719 (54.9)	318 (53.1)	100 (50.8)	301 (58.6)
Respiratory disease, n (%)				
No	280 (21.4)	165 (27.5)	51 (25.9)	64 (12.5)
Yes	1030 (78.6)	434 (72.5)	146 (74.1)	450 (87.5)
Hematologic_immune disease, n (%)				
No	1053 (80.4)	541 (90.3)	182 (92.4)	330 (64.2)
Yes	257 (19.6)	58 (9.7)	15 (7.6)	184 (35.8)
Infection, n (%)				
No	468 (35.7)	176 (29.4)	72 (36.5)	220 (42.8)
Yes	842 (64.3)	423 (70.6)	125 (63.5)	294 (57.2)
Surgery, n (%)				
No	875 (66.8)	297 (49.6)	110 (55.8)	468 (91.1)
Yes	435 (33.2)	302 (50.4)	87 (44.2)	46 (8.9)
Liver disease, n (%)				
No	673 (51.4)	295 (49.2)	101 (51.3)	277 (53.9)
Yes	637 (48.6)	304 (50.8)	96 (48.7)	237 (46.1)

SD = standard deviation.

Figure S1, https://links.lww.com/BS/A172, shows the distribution of APTT before logarithmic transformation (A) and after logarithmic transformation (B). The distribution was right-skewed before transformation, whereas the skewness was reduced after transformation, as indicated by decreases in both skewness (1.81 vs 0.89) and excess kurtosis (3.64 vs 0.64).

### 3.2. Model performances

**Figure 2.**
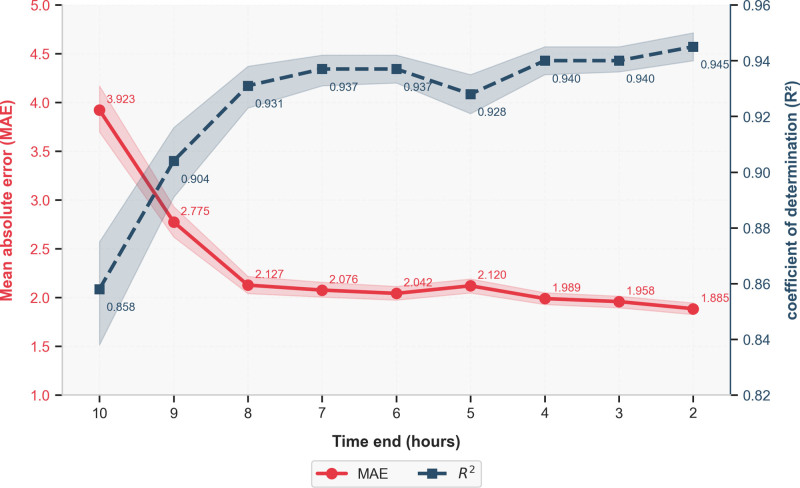
Model performance (mean absolute error and *R*^2^) across different prediction lead times. MAE = mean absolute error, *R*^2^ = coefficient of determination.

Table 2 presents model performance across different time windows, with results exponentiated back to the original scale. Table S1, https://links.lww.com/BS/A173, reports results on the log-transformed scale. The model using the [*t* − 12, *t* − 2] window achieved the best performance, with MAE, RMSE, *R*^2^, and MAPE of 1.885 (1.826–1.945), 4.732 (4.499–4.987), 0.945 (0.940–0.950), and 4.433% (4.328%–4.540%), respectively. When the prediction gap (lead time) was progressively decreased from 10 to 2 hours—that is, [*t* − 12, *t* − 10], [*t* − 12, *t* − 9], …, [*t* − 12, *t* − 2]—predictive performance improved gradually, particularly when the lead time was lower than 9 hours (**Fig. 2**).

**Table 2:** Model performance on the original-scale test set.

Window (h)	RMSE	MAE	*R* ^2^	MAPE
[*t* − 12, *t* − 10]	7.737 (7.136–8.344)	3.923 (3.697–4.170)	0.858 (0.838–0.875)	8.747 (8.354–9.169)
[*t* − 12, *t* − 9]	6.470 (6.011–6.983)	2.775 (2.621–2.933)	0.904 (0.891–0.916)	6.276 (6.001–6.546)
[*t* − 12, *t* − 8]	5.286 (4.966–5.629)	2.127 (2.041–2.217)	0.931 (0.923–0.938)	4.991 (4.829–5.142)
[*t* − 12, *t* − 7]	5.078 (4.828–5.352)	2.076 (2.005–2.157)	0.937 (0.931–0.942)	4.895 (4.749–5.026)
[*t* − 12, *t* − 6]	5.056 (4.830–5.300)	2.042 (1.974–2.114)	0.937 (0.932–0.942)	4.774 (4.640–4.900)
[*t* − 12, *t* − 5]	5.436 (5.127–5.717)	2.120 (2.047–2.191)	0.928 (0.921–0.935)	4.922 (4.788–5.060)
[*t* − 12, *t* − 4]	4.965 (4.750–5.198)	1.989 (1.929–2.050)	0.940 (0.935–0.945)	4.617 (4.502–4.738)
[*t* − 12, *t* − 3]	4.941 (4.720–5.154)	1.958 (1.895–2.014)	0.940 (0.936–0.945)	4.512 (4.393–4.607)
[*t* − 12, *t* − 2]	4.732 (4.499–4.987)	1.885 (1.826–1.945)	0.945 (0.940–0.950)	4.433 (4.328–4.540)

MAE = mean absolute error, MAPE = mean absolute percentage error, *R*^2^ = coefficient of determination, RMSE = root mean squared error.

**Figure 3.**
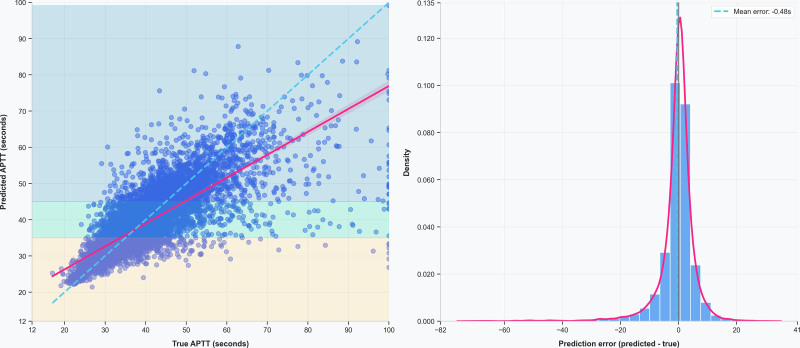
Performance of the APTT prediction model. (A) Comparison of predicted vs measured APTT values, with the line of perfect prediction and a fitted trend line; (B) distribution of the model’s prediction errors. APTT = activated partial thromboplastin time.

This pattern suggests that extending the prediction horizon reduces the temporal proximity between predictors and the target, thereby weakening temporal correlations and impairing model accuracy. Accordingly, the [*t* − 12, *t* − 2] model was selected for subsequent analyses. Figure 3A compares predicted APTT values with the true measurements, with a dashed blue line indicating the line of perfect prediction and a solid red line showing the actual trend. The data points are concentrated between 20 and 70 seconds, with some deviation from the perfect prediction line, suggesting strong predictive accuracy. In addition, the prediction errors are sharply centered around zero with a mean error of −0.48 seconds, suggesting minimal overall bias (**Fig. 3B**). The error distribution is narrow, indicating good model precision, with most errors falling close to zero. Overall, the model has good prediction performance. Figure S2, https://links.lww.com/BS/A172, shows three randomly selected individual trajectories of APTT alongside the corresponding predicted APTT values.

### 3.3. Subgroup analyses

**Figure 4.**
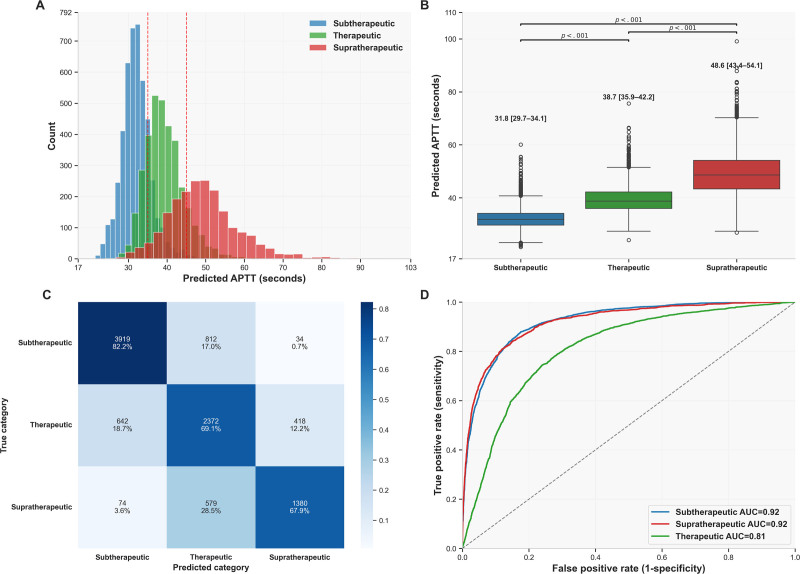
Categorization and evaluation of APTT predictions. (A) Distribution of predictions across subtherapeutic, therapeutic, and supratherapeutic ranges with clinical thresholds; (B) median predicted APTT values with confidence intervals for each therapeutic category; (C) confusion matrix comparing actual vs predicted therapeutic categories; (D) ROC curves evaluating the classification performance for different therapeutic states. APTT = activated partial thromboplastin time, AUC = area under the curve, ROC = receiver operating characteristic.

Subgroup analyses were conducted from multiple perspectives. First, the distribution of absolute prediction errors was examined within the critical range (APTT <35 or >45 seconds) and the noncritical range (APTT ∈ [35, 45] seconds). In both ranges, error distributions were sharply peaked near zero, with most absolute errors <10 seconds, indicating that most predictions were within an acceptable range (Figure S3A, https://links.lww.com/BS/A172). The error distribution in the critical range exhibited a slightly heavier right tail than that in the noncritical range, suggesting a modestly higher probability of larger errors when APTT values were outside the therapeutic range. Second, prediction errors were stratified by clinical risk: high-risk errors were defined as instances in which the observed APTT was outside the therapeutic range but the predicted value was within it; medium-risk errors were defined as instances in which the observed APTT was within the therapeutic range but the predicted value was outside it; and low-risk errors were defined as all other instances. As illustrated in Figure S3B, https://links.lww.com/BS/A172, among 10,230 predictions, 9731 (95.1%) were categorized as low risk, 349 (3.4%) as high risk, and 150 (1.5%) as medium risk, indicating that most predictions were associated with minimal clinical risk. Additionally, predictions were categorized as subtherapeutic (**Fig. 4A**, blue), therapeutic (green), or supratherapeutic (red), with vertical dashed lines denoting therapeutic boundaries. Subtherapeutic predictions clustered to the left of the therapeutic range, whereas supratherapeutic predictions clustered to the right. The median predicted APTT values were 31.8 seconds (IQR, 29.7–34.1) for subtherapeutic, 38.7 seconds (IQR, 35.9–42.2) for therapeutic, and 48.6 seconds (IQR, 43.4–54.1) for supratherapeutic predictions, with statistically significant differences among groups (*p* < .001; **Fig. 4B**). A confusion matrix comparing observed and predicted categories showed high classification accuracy: 82.2% for subtherapeutic, 69.1% for therapeutic, and 67.9% for supratherapeutic cases (**Fig. 4C**). ROC analyses further supported the model’s discriminative performance, with AUC values of 0.92 for distinguishing subtherapeutic from therapeutic states and 0.81 for distinguishing therapeutic from supratherapeutic states (**Fig. 4D**). Taken together, these results indicate that the model maintained robust performance across clinically relevant subgroups.

### 3.4. Clinical utility of the model

**Figure 5.**
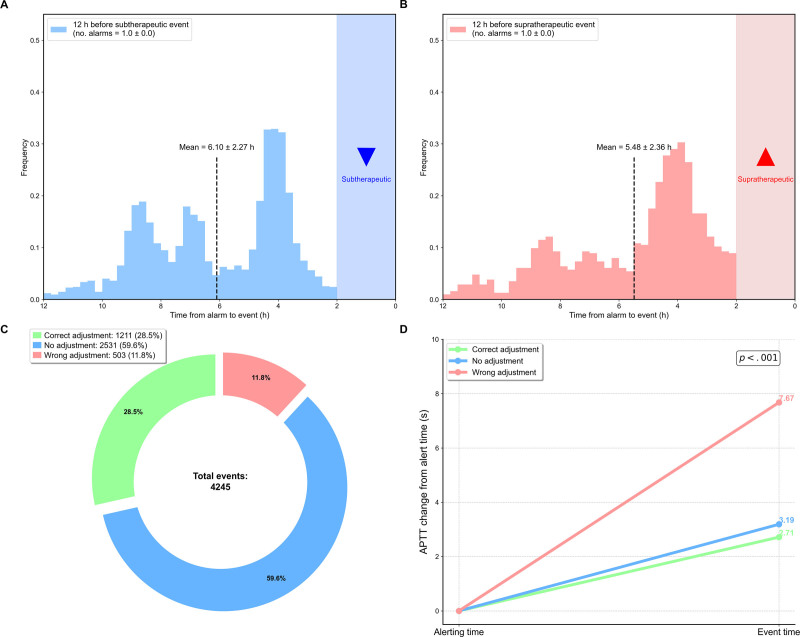
Early warning performance and clinical response analysis of the APTT prediction model. (A) Lead time for predicting subtherapeutic states; (B) lead time for predicting supratherapeutic states; (C) proportion of clinical responses following off-target predictions; (D) absolute APTT changes from alarm to event time across different response types. APTT = activated partial thromboplastin time.

The model demonstrated clinical utility by predicting impending subtherapeutic and supratherapeutic states an average of 6.10 ± 2.27 hours (**Fig. 5A**) and 5.48 ± 2.36 hours (**Fig. 5B**) in advance, respectively. Among APTT measurements outside the therapeutic range for which the model provided advance predictions, 59.6% were not followed by any heparin dose adjustment, 11.8% were followed by incorrect adjustments (eg, the model predicted a subtherapeutic state but the infusion rate was decreased, or vice versa), and 28.5% were followed by correct adjustments (**Fig. 5C**). In addition, the absolute change in APTT from the alarm time to the event time was greatest in the incorrect adjustment group (7.67 s) and smallest in the correct adjustment group (2.71 seconds; **Fig. 5D**). These findings indicate that the model provides early alerts for deviations from the therapeutic range and may offer improved guidance compared with physician-led management in approximately 70% of cases.

### 3.5. Feature importance analyses

**Figure 6.**
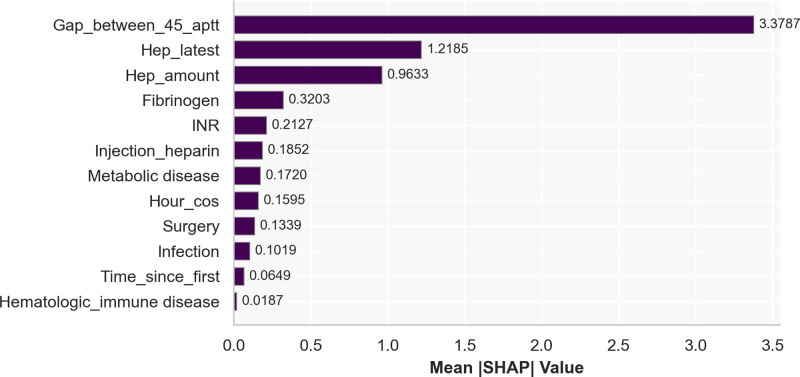
Feature importance analysis for the APTT prediction model. APTT = activated partial thromboplastin time, INR = international normalized ratio.

Feature importance analysis identified Gap_between_45_APTT as the most influential predictor. Specifically, larger Gap_between_45_APTT values were associated with higher predicted APTT values, indicating that the extent to which the most recent APTT exceeds the therapeutic upper limit of 45 seconds exerted the greatest influence on model predictions. Cumulative_heparin_dose and Heparin_infusion_rate ranked as the second and third most important predictors, respectively, underscoring the roles of cumulative heparin exposure and infusion intensity in determining APTT. Additional features that contributed substantially to the model included fibrinogen, INR, and intravenous heparin bolus dose (**Fig. 6**).

### 3.6. Model performance on the external validation dataset

As shown in Table 3 and Table S2, https://links.lww.com/BS/A173, the model demonstrated moderate-to-good predictive performance in the external validation dataset on both the original and log-transformed scales. On the original scale, RMSE ranged from 15.346–17.815, MAE ranged from 7.966–9.617, *R*^2^ ranged from 0.666 to 0.788, and MAPE ranged from 12.583%–16.254%. The best point estimates were observed for the [*t* − 12, *t* − 3] time window (RMSE, 15.346 [95% CI, 14.761–15.954]; MAE, 7.966 [95% CI, 7.589–8.370]; *R*^2^, 0.788 [95% CI, 0.776–0.800]; and MAPE, 12.583% [95% CI, 11.989–13.165]). Because the confidence intervals overlapped across several horizons, performance differences were modest and estimates were reasonably stable. Overall, the model produced consistent absolute errors of approximately 8 to 9 seconds and explained approximately 67% to 79% of the variance in APTT, supporting favorable generalizability. Table S3, https://links.lww.com/BS/A173, presents the performance of the linear regression and XGBoost models, which was lower than that of the LSTM model.

**Table 3:** Model performance on the original-scale validation set.

Window (h)	RMSE	MAE	*R* ^2^	MAPE
[*t* − 12, *t* − 10]	17.815 (14.590–20.810)	9.400 (7.420–11.665)	0.666 (0.579–0.755)	16.133 (12.555–20.282)
[*t* − 12, *t* − 9]	16.647 (14.656–18.719)	8.689 (7.479–9.975)	0.707 (0.650–0.763)	16.254 (14.025–18.519)
[*t* − 12, *t* − 8]	17.160 (15.773–18.586)	8.897 (7.930–9.864)	0.716 (0.686–0.745)	14.487 (13.008–15.962)
[*t* − 12, *t* − 7]	16.811 (15.741–17.905)	8.767 (8.059–9.551)	0.734 (0.711–0.754)	14.127 (13.115–15.246)
[*t* − 12, *t* − 6]	17.775 (16.977–18.730)	9.617 (9.002–10.336)	0.708 (0.687–0.726)	14.861 (14.058–15.817)
[*t* − 12, *t* − 5]	15.973 (15.254–16.765)	8.330 (7.864–8.834)	0.768 (0.748–0.785)	13.528 (12.791–14.329)
[*t* − 12, *t* − 4]	16.032 (15.379–16.769)	8.576 (8.140–9.057)	0.768 (0.753–0.781)	13.571 (12.848–14.321)
[*t* − 12, *t* − 3]	15.346 (14.761–15.954)	7.966 (7.589–8.370)	0.788 (0.776–0.800)	12.583 (11.989–13.165)
[*t* − 12, *t* − 2]	15.448 (14.867–16.032)	8.144 (7.714–8.564)	0.784 (0.771–0.796)	14.279 (13.487–15.172)

MAE = mean absolute error, MAPE = mean absolute percentage error, *R*^2^ = coefficient of determination, RMSE = root mean squared error.

## 4. DISCUSSION

The ability to predict APTT with high accuracy in real time has important clinical importance, particularly for optimizing heparin therapy in critically ill patients, for whom timely dose adjustments are essential.^15,16^ In this retrospective observational study, we developed and externally validated a deep learning model to predict future APTT values in critically ill patients with DVT receiving intravenous heparin. The model leveraged a comprehensive dataset including heparin administration data, laboratory test results, and medical history and achieved robust predictive performance across the full spectrum of APTT values. Notably, the model provided an average lead time of several hours for alerting clinicians to impending subtherapeutic or supratherapeutic APTT values. These findings support a shift in ICU heparin management from a reactive approach to a proactive, data–driven strategy, with the potential to enhance patient safety and treatment efficacy.

While two previous studies have reported models for predicting APTT, our investigation provides a distinct contribution to the field.^11,12^ Unlike previous study, which did not specifically focus on patients with DVT or incorporate heparin infusion data during model training, our study addressed these limitations by focusing on patients with DVT and integrating heparin infusion data into the training process. This approach more accurately reflects the clinical context of anticoagulation management in this population. Furthermore, we externally validated the model using a comprehensive dataset from the MIMIC-IV database, supporting its generalizability beyond the training environment. These methodological strengths enhance the value of this study for advancing models tailored to predicting APTT in patients with DVT. Another methodological advance was the systematic optimization of the input time window. Through testing and validation, we found that a look-back window spanning [*t* − 12, *t* − 2] hours yielded optimal performance. This window balances a longer historical context (12 hours) to characterize the patient’s trajectory with the exclusion of the most recent 2 hours to reduce transient noise, which appeared to improve predictive performance. This optimized window allows the model to remain informed by recent history while reducing sensitivity to short-term fluctuations, which is particularly important in dynamic ICU settings.

The robustness of the model was further supported by comprehensive subgroup analyses. The results indicated that the model maintained high predictive accuracy across critical (subtherapeutic and supratherapeutic) and noncritical (therapeutic) ranges, with particularly strong performance for values outside the therapeutic interval. This is clinically important because the primary value of prediction lies in identifying deviations from the target therapeutic window. In addition, the model demonstrated consistent performance across risk-based error categories, suggesting potential utility for reducing high-risk misclassifications associated with severe clinical consequences. To further evaluate discriminative performance, we conducted analyses using confusion matrices and ROC curves. These evaluations supported the model’s ability to distinguish among subtherapeutic, therapeutic, and supratherapeutic APTT categories. Notably, although performance remained robust overall, classification accuracy was slightly lower for supratherapeutic values. This finding may reflect the relative scarcity of observations in this range and the abrupt clinical interventions (eg, pausing the infusion) that often follow supratherapeutic values, which may introduce more irregular temporal patterns.

The model also demonstrated quantifiable clinical utility, particularly by providing advanced warning of impending subtherapeutic or supratherapeutic APTT values. This lead time provides an opportunity for proactive clinical decision-making. Rather than waiting for abnormal APTT results and then reacting, clinicians could use model outputs to consider preemptive dose adjustments, order confirmatory laboratory tests earlier, or increase monitoring frequency. Such proactive management may increase the time spent within the therapeutic range, which is closely associated with improved clinical outcomes in DVT treatment.^17,18^ Over the course of an ICU stay, this predictive capability may reduce the risks of both VTE progression and iatrogenic bleeding and may contribute to shorter hospital stays and lower healthcare costs.

To improve transparency and promote clinician trust, we conducted a feature importance analysis to clarify the basis of the model’s predictions. The results indicated that the model’s predictive patterns were consistent with established clinical principles. Notably, the difference between 45 seconds (the upper limit of the therapeutic APTT range) and the most recent APTT value, cumulative heparin dose, and heparin infusion rate were identified as the most influential predictors. In addition, the model assigned substantial importance to fibrinogen, INR, and intravenous heparin bolus dose, which are closely associated with coagulation dynamics. These findings suggest that the model integrates multiple aspects of patient status rather than relying solely on simplified dose–response relationships, thereby supporting a more physiologically informed assessment. Although interpretability tools may enhance clinician trust, the model is intended to complement, not replace, clinical expertise. This perspective aligns with broader discussions regarding the appropriate role of AI in healthcare, in which technology supports decision-making without supplanting clinicians’ nuanced judgment and patient-centered care. Despite strong performance on the internal test set, predictive accuracy declined modestly during external validation. Reduced performance in the MIMIC-IV cohort may reflect interinstitutional differences in laboratory assays, heparin dosing protocols, and monitoring frequency. Notably, the mean heparin infusion rate was substantially higher in the MIMIC-IV dataset than in the institutional dataset. Differences in patient populations across institutions, such as race and baseline thrombotic risk, may also affect model generalizability. Furthermore, discrepancies in data granularity (eg, broader chart abstraction intervals) may limit the model’s ability to capture relevant temporal patterns. These findings highlight the importance of standardizing data collection and clinical protocols across institutions when deploying predictive models to support heparin dosing in critically ill patients.

Through rigorous development and external validation, we introduced a promising tool that could improve the management of one of the most common and challenging therapies in critical care. While this study represents an important step forward, several limitations should be acknowledged. First, the retrospective nature of the data sources precludes establishing a causal relationship between model predictions and clinical outcomes. Future research should estimate the causal effect of adopting a model-informed heparin dosing strategy compared with standard care, for example, by using target trial emulation with observational data. Second, although the model was externally validated using the MIMIC-IV database, both the institutional dataset and the MIMIC-IV cohort were derived from large academic medical centers. Therefore, generalizability to community hospitals, diverse international settings with varying clinical practices, or specialized patient populations (eg, patients with heparin-induced thrombocytopenia) remains to be determined. Third, the model predicts a surrogate laboratory endpoint (APTT) rather than direct clinical outcomes, such as bleeding or thrombotic events. Although APTT is the standard measure for monitoring UFH therapy, future studies should evaluate whether model-guided management is associated with patient-centered outcomes. Fourth, the target APTT range in this study was 35 to 45 seconds, but this target may vary by institution, indication, or reagent. Future studies should evaluate the model using broader and adaptable target ranges. Finally, the inherent “black box” nature of deep learning remains a challenge for clinical implementation. Although feature importance analysis provides valuable insights into factors associated with the model’s predictions, the computations within the hidden layers remain largely opaque, which may limit full clinical acceptance. Despite these limitations, the model could be used as a clinical decision-support tool to assist clinicians in titrating heparin infusion rates for critically ill patients with DVT. The model is not intended to automate dosing decisions; rather, it is intended to support real-time monitoring and provide rapid, data-driven recommendations based on predicted APTT values. This approach may enhance clinical decision-making by providing actionable information tailored to individual patient trajectories while supporting safer and more efficient anticoagulant therapy in the ICU setting.

In conclusion, we developed and validated a deep learning model that accurately predicts future APTT values in critically ill patients with DVT receiving intravenous heparin. This study addresses an important gap in current practice by providing a data-driven approach that integrates temporal information and laboratory parameters to support proactive clinical intervention. The development of predictive models of this type represents an important advance in personalized medicine and highlights the potential of AI to enhance clinical decision-making in hematology and critical care. Although prospective validation is needed before widespread clinical adoption, this work provides a foundation for safer, more effective, and individualized anticoagulation therapy for patients with DVT.

## ACKNOWLEDGMENTS

This study was funded by the National High Level Hospital Clinical Research Funding 2025-PUMCH-A-200, Beijing Municipal Natural Science Foundation (Grant No. 7222134), CAMS Innovation Fund for Medical Sciences (CIFMS) (Grant No. 2025-I2M-C&T-A-001), and Scientific Research Promotion Program For Clinical Medicine (Grant No. 2026CMFA08).

The authors thank all the subjects for their participation in this study.

## ETHICAL APPROVAL

The ethics review board of Peking Union Medical College Hospital approved the study protocol (I-25PJ3176). Written informed consent of agreement on disclosure of desensitization information for non-profit and non-interventive clinical investigation was obtained from all patients or next-of-kin before the patients were admitted to the intensive care unit.

## AUTHOR CONTRIBUTIONS

S.L. and L.S. contributed to conception and design. Y.L. and W.Z. contributed to administrative support. B.T. and X.W. contributed to provision of study materials or patients. S.L. and A.G. contributed to collection and assembly of data. S.Z., A.G., S.L., and L.S. contributed to data analysis and interpretation. All authors contributed to manuscript writing and final approval of manuscript.

## Supplementary Material


