# Supplementary material for: Development and validation of a deep learning model to predict heparin response in critically ill patients with deep vein thrombosis receiving continuous intravenous heparin infusion
Source: Blood Sci. 2026 Aug 17;8(3):e00300. doi: 10.1097/BS9.0000000000000300 (PMC13485853; doi:10.1097/BS9.0000000000000300)

Figure S1. The distribution of APTT. A. Before logarithmic transformation; B. After logarithmic transformation. APTT, Activated Partial Thromboplastin Time

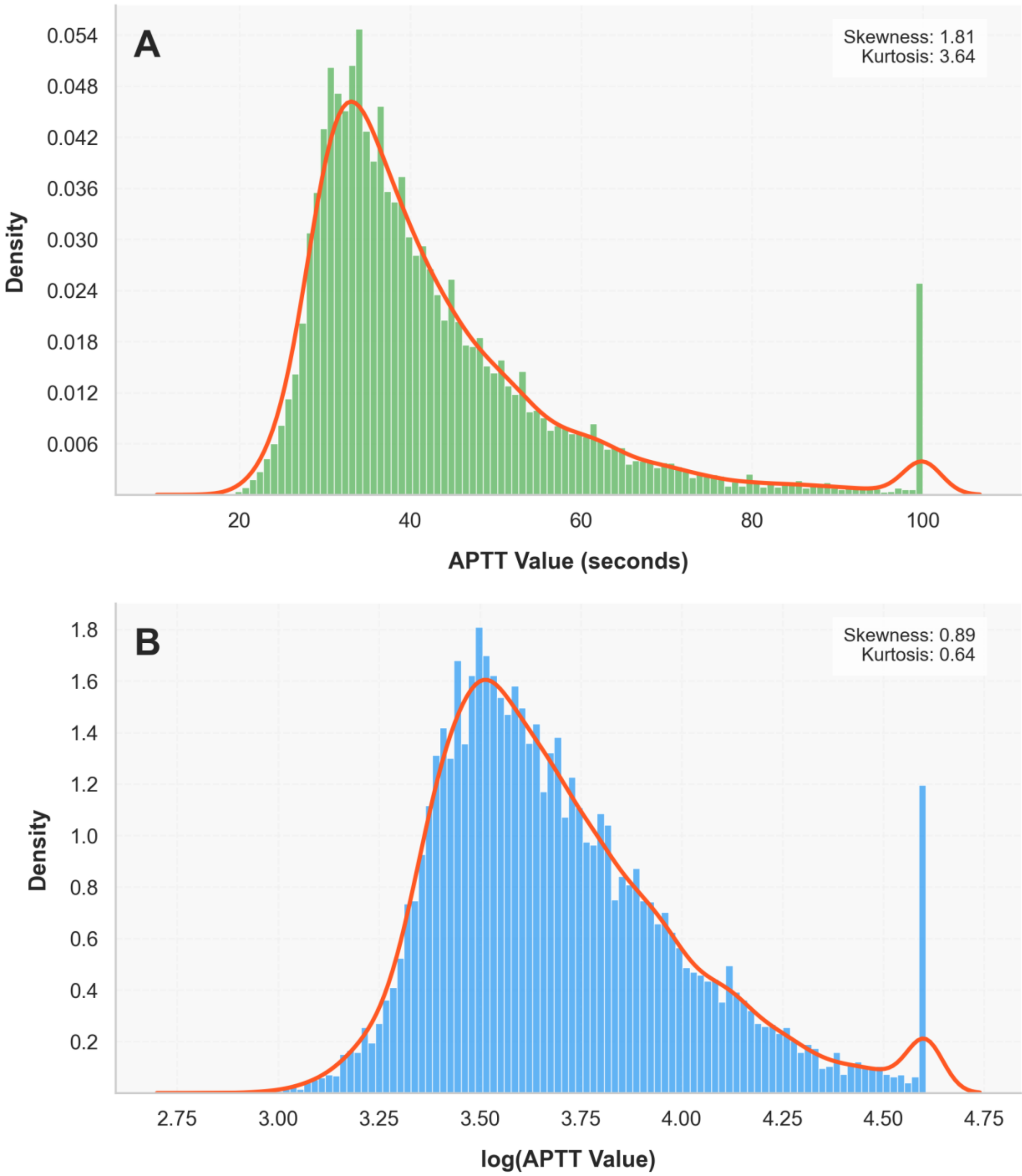

Figure S2. APTT trajectories for three randomly selected patients. APTT, Activated Partial Thromboplastin Time

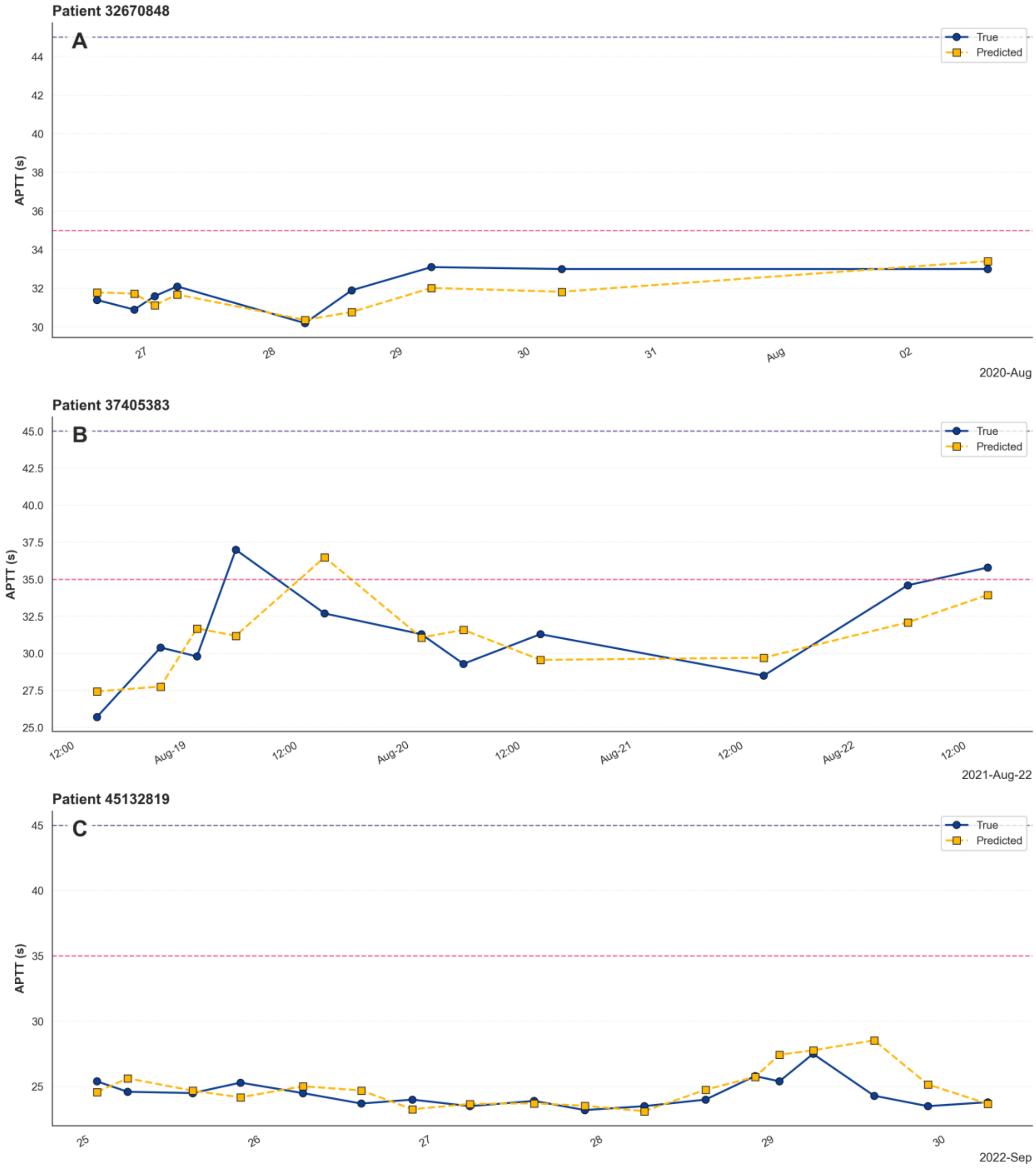

Figure S3. Prediction errors of the model. A. Distribution of absolute prediction error for critical zone and non-critical zone. B. Percentage of low-risk error, medium-risk error and high-risk error. APTT, Activated Partial Thromboplastin Time

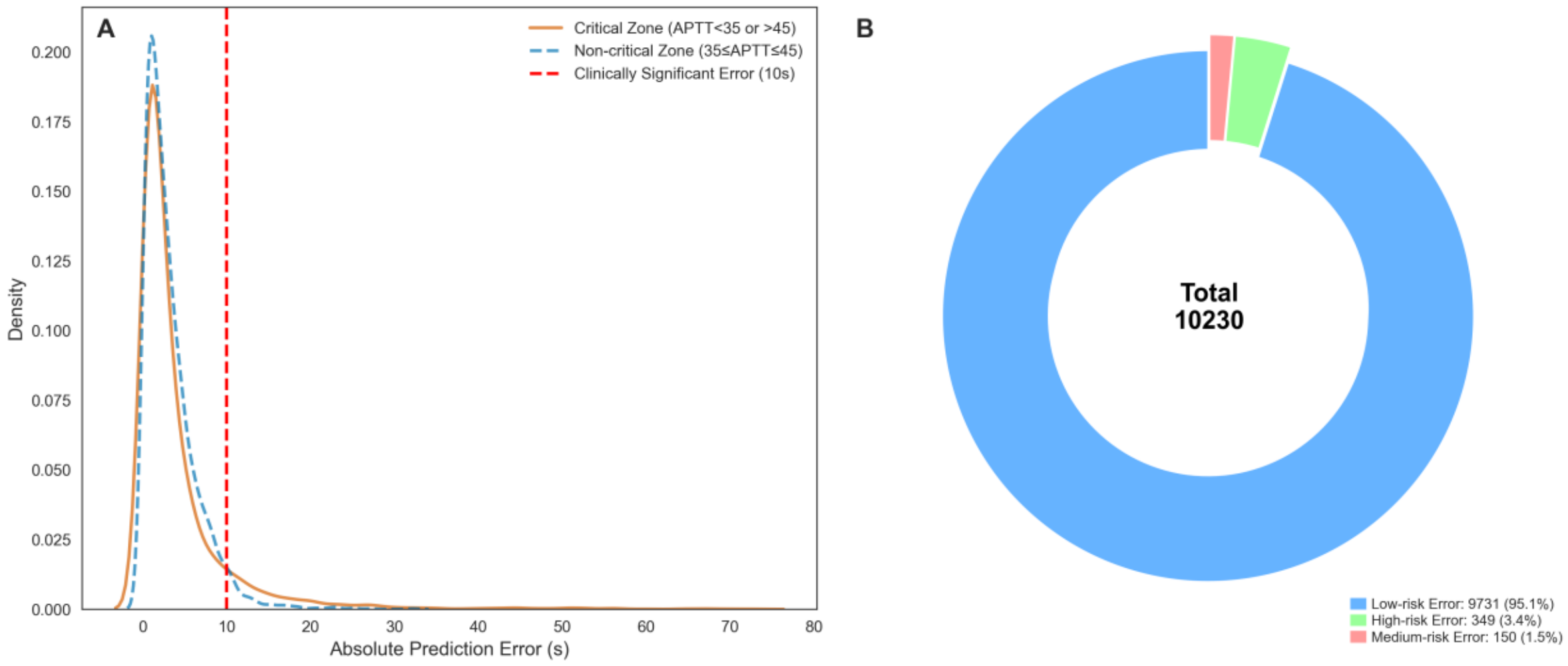

Supplement: Supplementary file 1 [file bs9-8-e00300-s001.pdf]
