# Supplementary material for: Development and validation of a deep learning model to predict heparin response in critically ill patients with deep vein thrombosis receiving continuous intravenous heparin infusion
Source: Blood Sci. 2026 Aug 17;8(3):e00300. doi: 10.1097/BS9.0000000000000300 (PMC13485853; doi:10.1097/BS9.0000000000000300)
Supplement: Supplementary file 2 [file bs9-8-e00300-s002.pdf]

Table S1 Model performance on the log-transformed test set

Window (h)	RMSE	MAE	R^2	MAPE
[t-12, t-10]	0.194 (0.154-0.237)	0.094 (0.088-0.100)	0.986 (0.980-0.991)	3.490 (3.300-3.721)
[t-12, t-9]	0.197 (0.158-0.237)	0.070 (0.065-0.075)	0.988 (0.983-0.992)	3.357 (3.148-3.587)
[t-12, t-8]	0.131 (0.112-0.150)	0.053 (0.051-0.055)	0.995 (0.993-0.996)	2.809 (2.716-2.902)
[t-12, t-7]	0.110 (0.103-0.118)	0.050 (0.049-0.052)	0.996 (0.996-0.997)	2.710 (2.641-2.779)
[t-12, t-6]	0.109 (0.103-0.117)	0.050 (0.048-0.051)	0.996 (0.996-0.997)	2.654 (2.588-2.717)
[t-12, t-5]	0.191 (0.169-0.213)	0.056 (0.053-0.059)	0.989 (0.986-0.991)	3.001 (2.881-3.148)
[t-12, t-4]	0.102 (0.098-0.105)	0.048 (0.046-0.049)	0.997 (0.997-0.997)	2.548 (2.500-2.602)
[t-12, t-3]	0.106 (0.100-0.113)	0.047 (0.046-0.048)	0.997 (0.996-0.997)	2.518 (2.454-2.574)
[t-12, t-2]	0.101 (0.095-0.109)	0.045 (0.044-0.047)	0.997 (0.996-0.997)	2.438 (2.386-2.489)

RMSE: Root Mean Squared Error, MAE: Mean Absolute Error, R^2: Coefficient of Determination, MAPE: Mean Absolute Percentage Error

Table S2 Model performance on the log-transformed validation set

Window (h)	RMSE	MAE	R^2	MAPE
[t-12, t-10]	0.419 (0.263-0.605)	0.188 (0.138-0.245)	0.956 (0.911-0.982)	9.210 (7.253-11.646)
[t-12, t-9]	0.442 (0.285-0.583)	0.179 (0.145-0.215)	0.951 (0.914-0.979)	8.919 (7.497-10.673)
[t-12, t-8]	0.291 (0.270-0.312)	0.161 (0.145-0.177)	0.979 (0.976-0.982)	7.838 (7.268-8.342)
[t-12, t-7]	0.278 (0.264-0.293)	0.155 (0.144-0.166)	0.981 (0.979-0.983)	7.525 (7.101-7.971)
[t-12, t-6]	0.300 (0.288-0.315)	0.173 (0.163-0.184)	0.978 (0.976-0.980)	8.282 (7.985-8.653)
[t-12, t-5]	0.369 (0.311-0.428)	0.155 (0.144-0.167)	0.967 (0.956-0.977)	7.518 (7.050-8.049)
[t-12, t-4]	0.260 (0.251-0.271)	0.148 (0.140-0.156)	0.984 (0.982-0.985)	7.135 (6.889-7.436)
[t-12, t-3]	0.248 (0.238-0.256)	0.135 (0.129-0.141)	0.985 (0.984-0.986)	6.539 (6.295-6.765)
[t-12, t-2]	0.263 (0.253-0.274)	0.146 (0.138-0.153)	0.983 (0.982-0.985)	6.740 (6.496-7.002)

RMSE: Root Mean Squared Error, MAE: Mean Absolute Error, R^2: Coefficient of Determination, MAPE: Mean Absolute Percentage Error

Table S3 Model performance using Linear regression or XGBoost regression on the original-scale

Model	Data Set	RMSE	MAE	R^2	MAPE
Linear regression	Test set	7.107 (6.783-7.411)	4.102 (3.991-4.205)	0.563 (0.536-0.592)	9.784 (9.598-9.956)
Linear regression	Validation set	75.319 (70.199-80.138)	36.899 (34.075-39.896)	-11.246 (-12.913-9.752)	60.536 (54.857-65.942)
XGBoost	Test set	7.199 (6.900-7.482)	4.279 (4.164-4.384)	0.551 (0.528-0.573)	10.153 (9.933-10.352)
XGBoost	Validation set	23.181 (22.352-23.930)	17.428 (16.753-18.060)	-0.160 (-0.219-0.104)	27.887 (26.874-28.855)

RMSE: Root Mean Squared Error, MAE: Mean Absolute Error, R^2: Coefficient of Determination, MAPE: Mean Absolute Percentage Error
